# *Porphyromonas gingivalis* can invade periodontal ligament stem cells

**DOI:** 10.1186/s12866-017-0950-5

**Published:** 2017-02-17

**Authors:** Chunling Pan, Junchao Liu, Hongyan Wang, Jia Song, Lisi Tan, Haijiao Zhao

**Affiliations:** Department of Periodontics, School of Stomatology, China Medical University, Shenyang, 110002 China

**Keywords:** *Porphyromonas gingivalis*, Periodontal ligament stem cells, Periodontitis

## Abstract

**Background:**

*Porphyromonas gingivalis* is strongly associated with the development, progression, severity and recurrence of periodontitis. Periodontal ligament stem cells (PDLSCs) play an important role in the maintenance of periodontal tissue self-renewal and repair. The purpose of this study was to investigate the ability of *P. gingivalis* to infect PDLSCs using an in vitro monolayer model.

**Methods:**

We separated and cultured primary PDLSCs using the tissue block with limiting dilution method. The efficiency of *P. gingivalis* (ATCC 33277) infection of PDLSCs was measured using agar plate culture and quantitative polymerase chain reaction (q-PCR) methods. PDLSCs infected with *P. gingivalis* were also observed by transmission electron microscopy.

**Results:**

We assessed stem cell properties including cell morphology, clone formation, growth activity, cell surface antigens and multiple differentiation capacity. The infection rates of *P. gingivalis* in PDLSC at MOIs of 50, 100, 200, and 500 were 5.83%, 8.12%, 7.77% and 7.53% according to the agar plate culture method. By q-PCR, the efficiencies of *P. gingivalis* infection of PDLSCs at MOIs of 50, 100, 200, and 500 were 6.74%, 10.56%, 10.36% and 9.78%, respectively. Overall, the infection efficiency based on q-PCR was higher than that according to agar plate culture. Using transmission electron microscopy, we verified that *P. gingivalis* (ATCC 33277) could infect and invade PDLSCs after 2 h of incubation, and endocytic vacuoles were not found surrounding the internalized bacteria.

**Conclusions:**

In conclusion, our data demonstrate that *P. gingivalis* can invade PDLSCs.

**Electronic supplementary material:**

The online version of this article (doi:10.1186/s12866-017-0950-5) contains supplementary material, which is available to authorized users.

## Background

Periodontitis, a chronic infectious disease in periodontal support tissues that can induce or aggravate multi-system diseases such as diabetes [1], cardiovascular disease [2] and chronic obstructive pulmonary disease [3], has become one of the most common human diseases causing damage to both oral and general health. Periodontitis also induces a local immune inflammatory response, which results in the irreversible destruction of periodontal attachment and the loss of periodontal regeneration, eventually leading to tooth extraction. It is well known that plaque and their products are the initial factors of periodontitis [4].

Large numbers of bacteria reside in the oral cavity, including *Porphyromonas gingivalis*, a gram-negative anaerobic bacterium. The virulence factors of *P. gingivalis*, such as lipopolysaccharide, vesicles, gingipains and fimbriae, not only directly destroy periodontal tissue but also cause secondary tissue damage by producing an inflammatory response [5]. Furthermore, *P. gingivalis* develops a dynamic balance and symbiosis with the host tissue in which it resides, enabling the bacteria to evade the host immune response [6]. Therefore, *P. gingivalis* is considered to be an important periodontal pathogen and has a close relationship with the development, progression, severity and recurrence of periodontitis [7].

Although periodontitis can be controlled through conventional therapies, it is difficult to restore damaged periodontal structures [8]. Indeed, the goal of periodontal treatment is to recover periodontal function by rebuilding the attachment tissue. However, during infection, bacteria can alter the surrounding environment and inhibit endogenous cell differentiation [9]. In addition, chronic inflammation can inhibit tissue repair by reducing cell proliferation and migration, two of the most important factors in this process. Earlier studies have found that stem cells play a crucial role in maintaining normal tissue regeneration and promoting repair of damaged tissue. In 2004, Seo et al. successfully separated and identified a type of adult stem cell from the periodontal membrane [10]. These stem cells, named periodontal ligament stem cells (PDLSCs), exhibit strong proliferation, clone formation and multi-directional differentiation capacities after proper induction in vitro. In fact, PDLSCs can differentiate into mature periodontal fibroblasts, cementoblasts and osteoblasts to achieve complete periodontal tissue self-renewal to repair defects [11]. The dynamic balance and differentiation capabilities of PDLSCs are the biological basis for periodontal regeneration and repair, and as a result, PDLSCs play an important role in the maintenance of periodontal tissue self-renewal and repair [12].

Recently, researchers have confirmed that *P. gingivalis* can invade gingival epithelial cells and disrupt the epithelial barrier to infect deeper tissues [13]. The intracellular bacteria can then alter cellular functions, including migration, cell cycle progression [14, 15] and apoptosis [16]. Additionally, *Fusobacterium nucleatum* and *Candida albicans* are able to enhance the adhesion and invasion of *P. gingivalis* and restrain the host innate immune response to exacerbate infection [17–19]. Bacteria located inside cells are considered to have “escaped” from host immune surveillance and antibiotic pressure, leading to intracellular persistence, multiplication, and dissemination to adjacent tissues. The process by which *P. gingivalis* invades cells is divided into four phases: adhesion, entry, intracellular traffic and exit [20]. To enter and exit host cells, *P. gingivalis* exploits the cellular endocytosis pathway, which leads to persistent tissue infection [21].

It is crucial to understand the interaction between *P. gingivalis* and PDLSCs in the development of periodontitis and ensuing tissue repair. However, there are few published studies examining this relationship. Therefore, the purpose of this study was to investigate the ability of *P. gingivalis* to infect PDLSCs using an in vitro monolayer model.

## Methods

### Culture and isolation of PDLSCs

The protocol in the present study was approved by the ethics committee of the School of Stomatology of China Medical University (G2014010). Each participant (parent or legal guardian in the case of participant under 18 years of age) provided written informed consent to donate his or her extracted teeth prior to enrollment. Human periodontal ligament tissues from twenty healthy individuals (twelve males and eight females, aged 12 to 30 years old) were obtained from healthy premolars extracted for orthodontic reasons. The subjects included in the study had no history of systemic diseases or periodontal treatment and had not recently taken antibiotics. PDLSCs were cultured using the tissue block with limiting dilution technique. In brief, the extracted teeth were placed immediately in Dulbecco’s Modified Eagle’s Medium (L-DMEM) (Hyclone Laboratories Inc., South Logan, UT, USA) with 200 U/ml penicillin and 200 mg/ml streptomycin (Hyclone Laboratories Inc., South Logan, UT, USA). After the teeth were rinsed, the human periodontal ligament tissues on the middle third of the root surfaces were collected and cut into 1-mm^3^ pieces. The minced tissues were plated in 6-well culture plates (Costar, Corning Inc., NY, USA) and cultured with L-DMEM medium supplemented with 15% fetal bovine serum (FBS) (Hyclone Laboratories Inc., South Logan, UT, USA) with 100 U/ml penicillin and 100 mg/ml streptomycin (Hyclone Laboratories Inc., South Logan, UT, USA) in a humidified atmosphere with 5% CO_2_ at 37 °C. The human periodontal ligament cells were at 70% confluence for 2–3 weeks. To further separate and purify PDLSCs, a single-cell suspension was obtained and diluted to 10 cells/ml, and individual cells were seeded in a well of a 96-well plate with 100 μl basal medium (L-DMEM medium supplemented with 10% FBS, 100 U/ml penicillin and 100 mg/ml streptomycin). Wells that included a single cell were marked after cultivation of 12 h. All colonies were collected and pooled as PDLSCs (passage 1). To avoid a significant loss of phenotype caused by prolonged culture, the PDLSCs were used at passages 2–6 for all of the following experiments.

### The colony-forming ability of PDLSCs

A total of 2000 PDLSCs were plated 1 in a 10-cm-diameter culture dish containing basal medium and cultured with the addition of fresh medium for 15 days. After cell clusters formed, the cells were fixed with methanol for 20 min, rinsed twice with phosphate-buffered saline (PBS), and stained with Giemsa for 30 min. Colony-forming units (CFUs) in which there were more than 50 cells were counted and observed under a microscope. This experiment was repeated three times.

### Immunohistochemical staining of PDLSCs

PDLSCs were seeded on a slide in 6-well culture plates. After 2 days of incubation, the samples were fixed in 4% paraformaldehyde for 60 min. The fixed cells were treated with 0.1% Triton X-100 for 5 min, blocked for 1 h, and incubated overnight with the selected primary antibody against vimentin or keratin (Santa Cruz Biotechnology Inc., CA, USA) at 4 °C. The subsequent steps were performed according to the standard protocol of the biotin-streptavidin in staining kit (Santa Cruz Biotechnology Inc., CA, USA), and the slides were observed under a microscope.

### PDLSCs growth curve and cell cycle

The growth curve of PDLSCs was analyzed using the CCK-8 assay (Beyotime Biotechnology Inc., Shanghai, China). In brief, PDLSCs were plated at a density of 2×10^3^ cells/well with 100 μl basal medium in a 96-well plate. At the same time each day for 10 days consecutively, 10 μl CCK-8 solution was added to the wells and then incubated for 2 h at 37 °C. Absorbance was measured at 450 nm using a microplate reader (Tecan Group Ltd., Seestrasse, Switzerland).

The cell cycle of PDLSCs was examined by flow cytometry. Cells were inoculated in a cell flask with basal medium. Cells at 70% confluence were digested with trypsin and then fixed overnight in 70% ice-cold dehydrated ethanol at 4 °C. The fixed cells were gently rinsed twice with PBS, stained with propidium iodide (Beyotime Biotechnology Inc, Shanghai, China) for 30 min in the dark on ice and analyzed by flow cytometry (Becton, Dickinson and Company, NJ, USA). The proportions of cells remaining in G0/G1 phase, S phase, and G2/M phase were calculated.

### Cell surface antigen expression on PDLSCs

Expression of cell surface markers of PDLSCs was determined by flow cytometry. Approximately 1×10^6^ PDLSCs were collected and suspended in PBS with 0.5% bovine serum albumin (BSA) and then incubated with fluorescein isothiocyanate (FITC) -conjugated monoclonal antibodies against human CD45, CD34, CD90, CD29, CD146, CD105 (Abcam plc, Cambridge, UK) and Stro-1 (Santa Cruz Biotechnology Inc., CA, USA) for 30 min on ice in the dark. The cells were then washed twice with cold PBS containing 0.5% BSA. The isotype antibody was used as a control. The cells with labeled surface antigens were analyzed using a flow cytometer (Becton, Dickinson and Company, NJ, USA).

### The multi-potent differentiation ability of PDLSCs in vitro

The multi-potent differentiation ability of PDLSCs was investigated. PDLSCs were plated in a 6-well plate at a density of 2×10^4^/well with basal medium. When the cells reached 80% confluence, the medium was replaced with differentiation medium. The subsequent steps were performed following the standard protocol for human mesenchymal stem cell osteogenic differentiation medium or human bone marrow mesenchymal stem cell adipogenic differentiation medium (Cyagen Biosciences Inc, Guangzhou, China). The fresh medium was changed twice per week for 3 weeks. Finally, the mineralized nodules were dyed with 2% alizarin red, and the lipid droplets were stained with oil red O according to the manufacturer’s protocol. The cells were observed and images recorded using a microscope.

### *P. gingivalis* culture and PDLSCs infection

*P. gingivalis* (ATCC 33277) was used to evaluate its invasion capacity. The Department of Oral Biology at China Medical University maintains a frozen stock of *P. gingivalis* ATCC 33277, which was originally obtained from American Type Culture Collection. The bacteria were routinely grown in brain heart infusion (BHI) blood agar medium or BHI broth supplemented with 0.5% yeast extract, hemin (10 μg/ml), and vitamin K (1 μg/ml). A bacterial suspension was incubated overnight and collected by centrifugation at 5000xg at 4 °C. After two washes with PBS, the cells were resuspended in L-DMEM without antibiotics, and the bacterial density was allowed to reach an OD of 1.0 at 600 nm.

The PDLSCs were inoculated into 6-well plates with 5×10^5^ cells per well with basal medium without antibiotics for 24 h. *P. gingivalis* ATCC 33277 was added according to the number of PDLSCs, and the multiplicity of infection (MOI) listed following cell counts was determined using a hemocytometer.

### Infected CFUs of *P. gingivalis* ATCC 33277 according to the agar plate culture method

Non-adherent bacteria were removed by 5 rinses after infection for 2 h, and then the PDLSCs infected with *P. gingivalis* ATCC 33277 were lysed with sterile distilled water for 30 min. Finally, the released bacteria were inoculated onto blood agar plates and cultured for 7 days under anaerobic conditions. The CFUs of the bacteria were counted, and infection is expressed as the percentage of the initial inoculum recovered after PDLSC lysis.

### The infection ability of *P. gingivalis* ATCC 33277 according to the q-PCR method

A gradient dilution suspension of *P. gingivalis* ATCC 33277 from 10 to 10^8^/ml was prepared. After the bacteria were centrifuged at 5000xg for 5 min, DNA was extracted from the cell pellet according to the manufacturer’s protocol (Biomed Ltd, Beijing, China). The q-PCR was carried out with primers specific for *P. gingivalis*: 16S (forward: 5’-ACCTTACCCGGGATTGAAATG-3’; reverse: 5’-CAACCATGCAGCACCTACATAGAA-3’). A standard curve was generated using the CT value threshold as the ordinate and the number of bacteria as the abscissa (Additional file 1: Figure S1). After PDLSCs were infected with *P. gingivalis* ATCC 33277 for 2 h, the cells were washed 5 times to remove unattached bacteria, digested with trypsin and centrifuged at 1000x g for 5 min. The collected pellets were used to extract bacterial DNA for q-PCR. The number of bacteria was calculated according to the standard curve.

### Electron microscopy examination of PDLSCs infected with *P. gingivalis* ATCC 33277

PDLSCs were grown to near 50% confluency, and *P. gingivalis* ATCC 33277 was added to the dish until the MOI reached 100. After incubation for 2 h, the monolayer cells were washed 5 times with PBS and treated with trypsin. Detached cells were collected in tubes and fixed in 2.5% glutaraldehyde. The samples were then postfixed, dehydrated in graded acetone, and embedded. Thin sections of the specimens were cut and viewed and photographed using an electron microscope.

### Statistical analysis

All experiments were performed in triplicate for each condition and repeated at least three times. The independent-sample t-test was used to analyze differences between the two groups. The data were analyzed with ANOVA in a group. *P* values of less than 0.05 were considered to be statistically significant.

## Results

### Culture, isolation and identification of PDLSCs

**Fig. 1:**
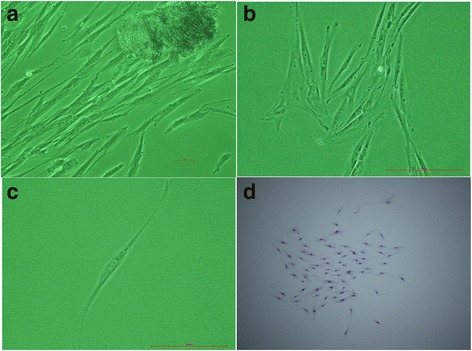
Periodontal ligament cells were cultured, and periodontal ligament stem cells were separated and cloned. The periodontal ligament cells emerged from the tissue on the 7th day (**a**). The periodontal ligament cells reached 100% confluence after 20 days (**b**). We separated and purified periodontal ligament stem cells using the limiting dilution method. A single cell was marked after cultivation for 12 h (**c**). The cloned periodontal ligament stem cells were closely arranged and colony-like. The cells were spindle shaped and polygonal (**d**)

We successfully cultured original periodontal ligament cells using the tissue block method. The cells exhibited static adherence around the tissue block on the 7th day and were fusiform. With extension of the incubation time, the cells gradually increased and were 70% confluent after approximately 20 days. The periodontal ligament cells exhibited a long spindle or polygonal shape; the cytoplasm was abundant and contained round or ovoid nuclei, as viewed under by microscopy (Fig. 1a and b).

Many studies have shown that periodontal ligament cells comprise a heterogeneous group that includes osteoblasts, fibroblasts and stem cells, among others. To further separate and purify PDLSCs, a single-cell clone was formed using the limiting dilution method. The cloned PDLSCs were closely arranged and colony-like. The cells were spindle shaped and polygonal. The cloning efficiency of PDLSCs in our study was approximately 15.35% (Fig. 1c and d).

**Fig. 2:**
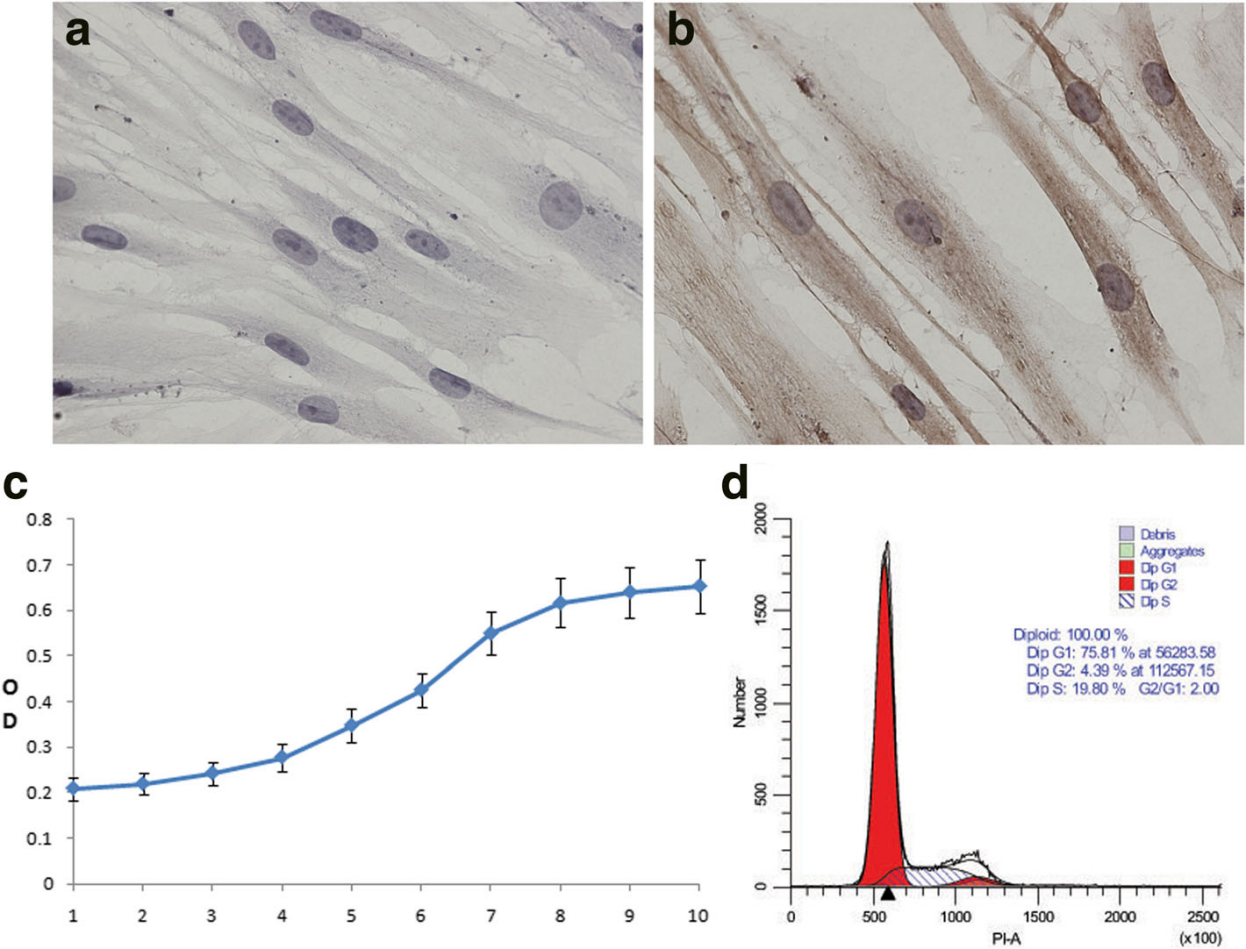
The properties and characteristics of periodontal ligament stem cells. The cells were negative for keratin expression according to immunohistochemical staining (40X) (**a**). The cells were positive for vimentin expression. Several brown particles in the cytoplasm were observed (40X) (**b**). Growth was measured at passage 2. The growth curve of PDLSCs exhibited an “S” shape (**c**). The cell cycle of PDLSCs was examined at passage 5, showing that most cells were in G1 phase; proliferation was slow (**d**)

The results from immunohistochemical staining revealed positivity for vimentin, indicated by brown particles in the cytoplasm, but negativity for keratin (Fig. 2a and b). Therefore, the PDLSCs were derived from the mesoderm.

As one of the basic parameters of biological characteristics, a cell growth curve is typically used to evaluate viability. In our study, the PDLSC growth curve was measured at passage 2. After the cells were in the latent phase from the 1st day to the 3rd day, they began to grow quickly and were in the logarithmic phase from the 4th day to the 8th day. The cells then reached the growth peak and were in the stationary phase on the 9th and 10th days (Fig. 2c).

The cell cycle of PDLSCs in the 5th generation was measured, revealing that most of the cells were in the stationary phase and the early stage of DNA synthesis (75.81%). The cells in G2 phase accounted for 4.39% and those in S phase for 19.80% (Fig. 2d).

**Fig. 3:**
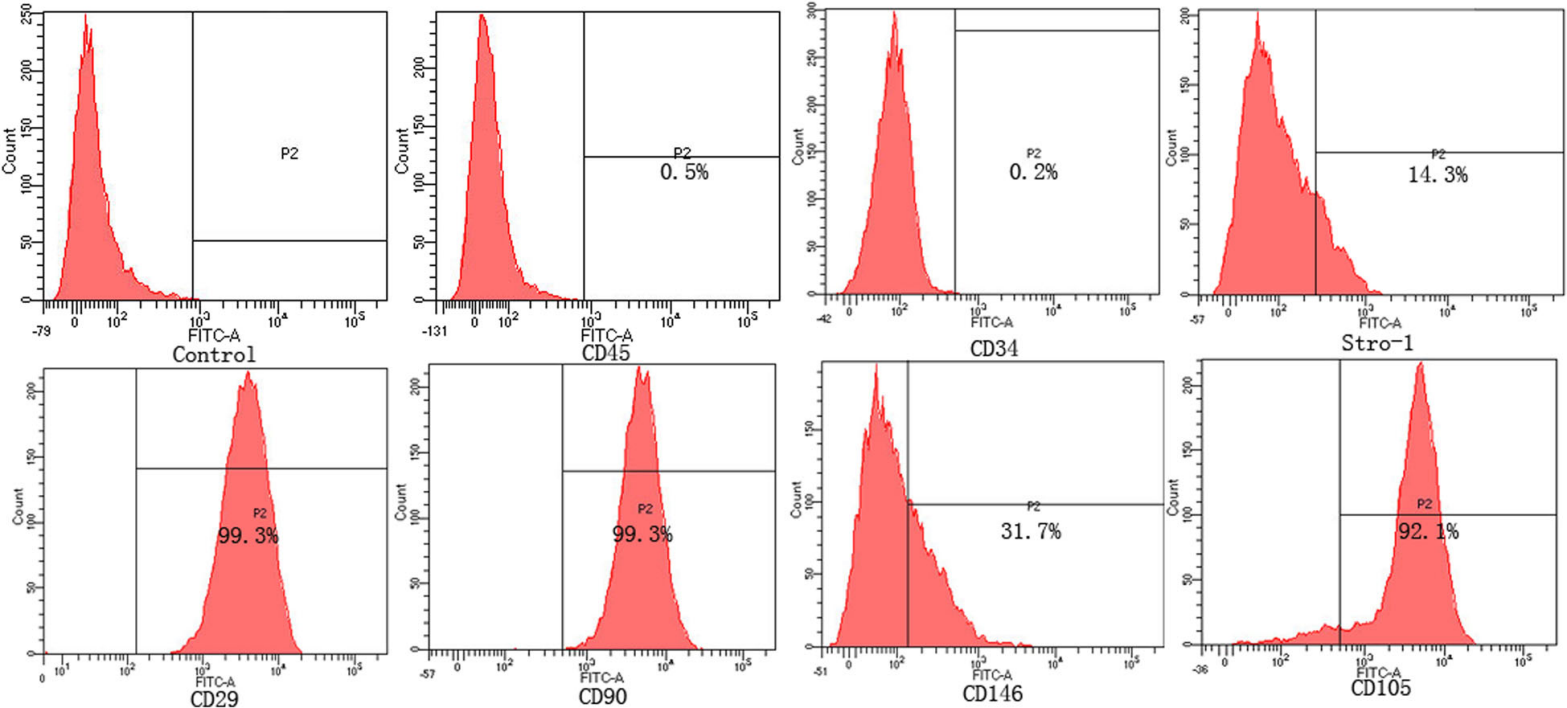
Cell-surface antigens expressed on PDLSCs. PDLSCs were positive for specific surface markers of mesenchymal stem cells (CD29, CD90, CD105, CD146, STRO-1) and negative for those of hematopoietic cells (CD34, CD45). The isotype antibody was used as a control

The results of the flow cytometric analysis for molecular markers showed that the PDLSCs were positive for specific mesenchymal stem cell surface markers (CD29, CD90, CD105, CD146, STRO-1) and negative for those of hematopoietic cells (CD34, CD45), which proved that the cells possess properties of stem cells (Fig. 3).

**Fig. 4:**
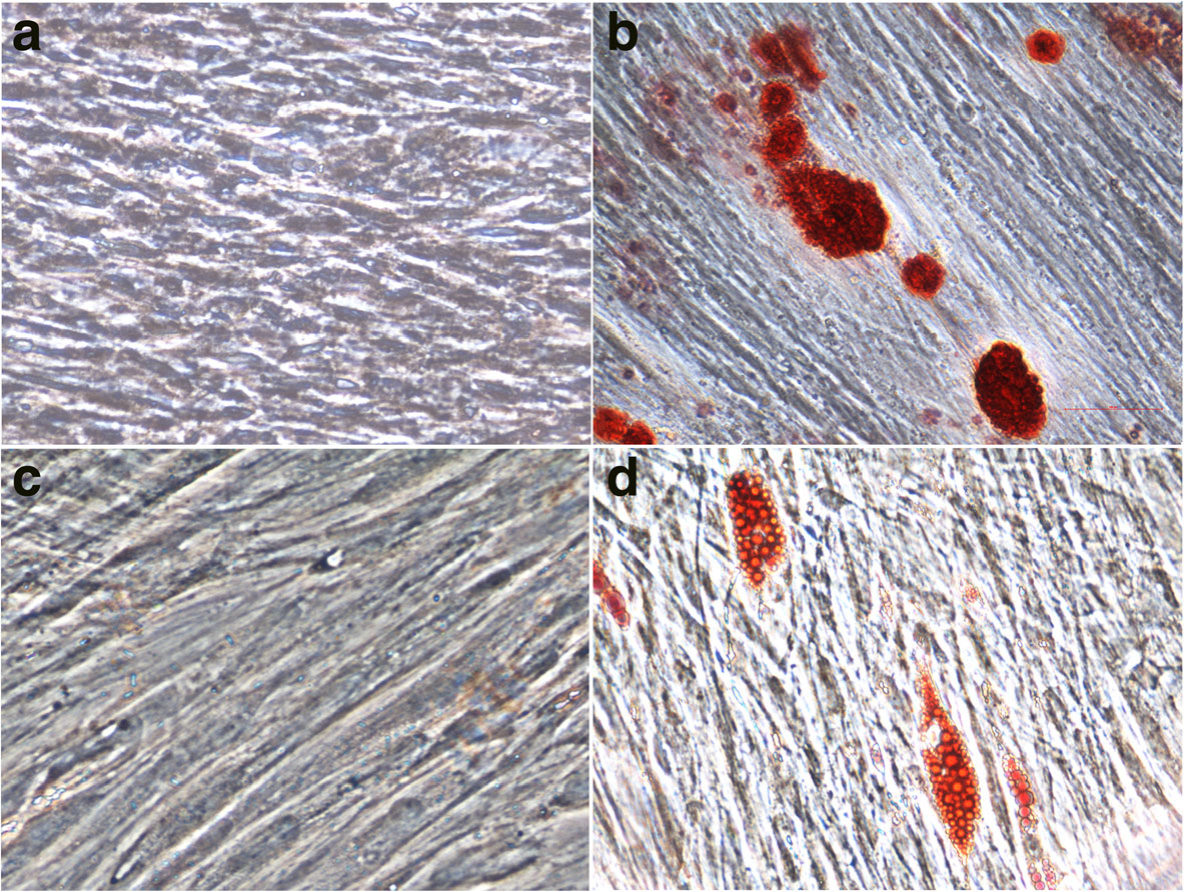
The multi-directional differentiation capacity of PDLSCs. There were no visible mineralized nodules in the PDLSCs using alizarin red staining before osteogenesis induction (Fig. 4a). Alizarin red staining revealed many mineralized nodules after 14 days of induced osteogenesis (Fig. 4b). The lipid droplets did not observed by oil red O staining without adipogenic induction (Fig. 4c). Oil red O staining revealed a large number of lipid droplets in the cytoplasm after adipogenic induction for 20 days (Fig. 4d)

There were no visible mineralized nodules in PDLSCs using alizarin red staining before osteogenesis induction (Fig. 4a). After 14 days of osteogenesis induction, alizarin red staining showed mineralized nodules in the PDLSCs (Fig. 4b). The lipid droplets did not observed by oil red O staining without adipogenic induction (Fig. 4c). After 2 weeks of adipogenic differentiation, the morphology, as determined by microscopy, of the PDLSCs changed. After adipogenic induction for 20 days, oil red O staining revealed a large number of lipid droplets in the cytoplasm (Fig. 4d).

### The efficiency of *P. gingivalis* ATCC 33277 infection of PDLSCs

**Fig. 5:**
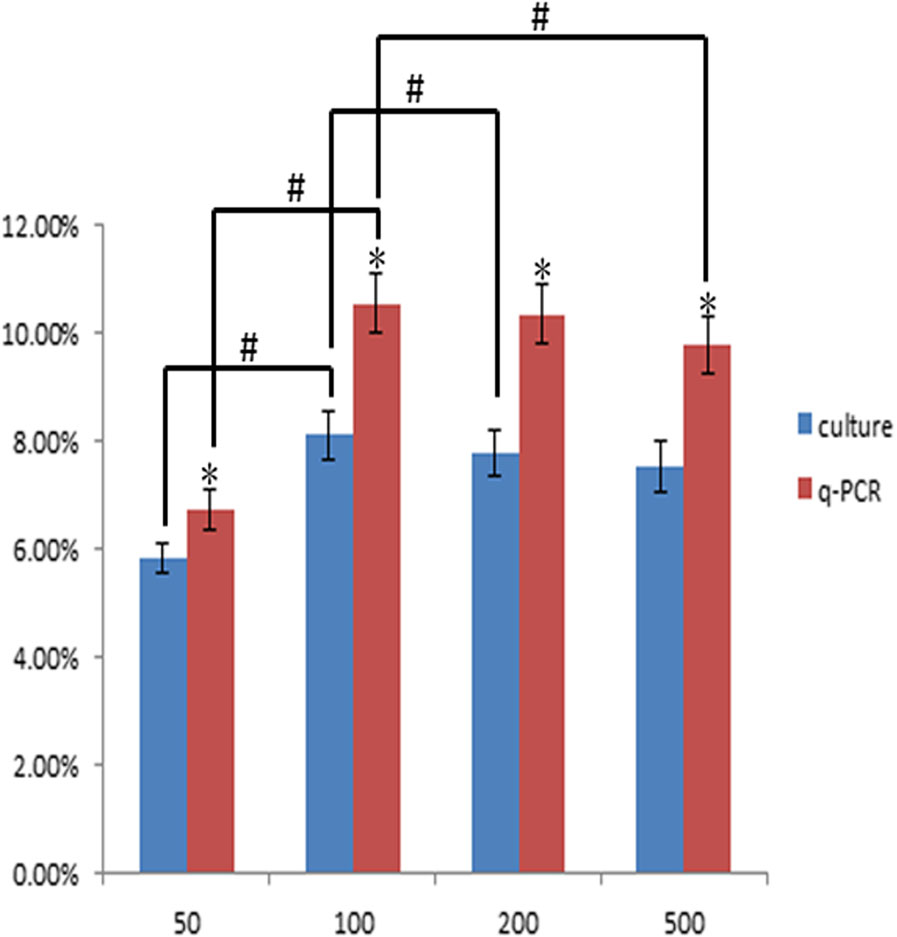
The efficiency of *P. gingivalis* ATCC 33277 infection in PDLSCs. The infection rate of *P. gingivalis* in PDLSCs at MOIs of 50, 100, 200, and 500 were 5.83%, 8.12%, 7.77% and 7.53%, respectively, according to the agar plate culture method. The efficiencies of *P. gingivalis* infection of PDLSCs at MOIs of 50, 100, 200, and 500 were 6.74%, 10.56%, 10.36% and 9.78%, respectively, according to the q-PCR method. The data are presented as the mean ± SD of triplicate experiments. *Significant difference (*P* < 0.05) compared with agar plate culture method and q-PCR method. # Significant difference (*P* < 0.05) compared with different MOIs in a group

The infection efficiency of *P. gingivalis* ATCC 33277 in PDLSCs was measured using agar plate culture and q-PCR methods. The PDLSC infection rate of *P. gingivalis* at MOIs of 50, 100, 200, and 500 were 5.83%, 8.12%, 7.77% and 7.53%, respectively, according to the agar plate culture method. By q-PCR, the efficiencies of *P. gingivalis* infection of PDLSCs at MOIs of 50, 100, 200, and 500 were 6.74%, 10.56%, 10.36% and 9.78%, respectively. The infection efficiency according to q-PCR method was higher than that according to the agar plate culture method at any MOI, a difference was significant (Fig. 5). The infection efficiency of *P. gingivalis* ATCC 33277 in PDLSCs at MOI of 50 was lower than that at any other MOIs by agar plate culture and q-PCR methods, there were statistically significant differences. The infection rate at an MOI of 100 was the highest, which were statistical differences with that at MOIs of 200 and 500 by agar plate culture method and significant differences with that at an MOI of 500 by q-PCR method. There *Escherichia coli* DH5a was used as a control, showing a less than 0.001% infection rate of PDLSCs at MOIs from 50 to 500.

### PDLSCs infected with *P. gingivalis* ATCC 33277 under transmission electron microscopy

**Fig. 6:**
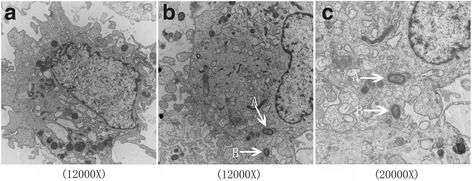
PDLSCs infected with *P. gingivalis* ATCC 33277 under transmission electron microscopy. The nucleus was large and round, and organelles were abundant in PDLSCs (**a**). *P. gingivalis* ATCC 33277 could invade PDLSCs after 2 h of incubation (**b**, **c**-*A*). Endocytic vacuoles were not found surrounding internalized *P. gingivalis*. The bumps observed were stretched membrane where the PDLSCs packaged *P. gingivalis* ATCC 33277 (**b**, **c**-*B*)

Abundant organelles, such as mitochondria, the endoplasmic reticulum, and the Golgi apparatus, were found in the cytoplasm of normal PDLSCs, and the nucleus was large and round (Fig. 6a). *P. gingivalis* ATCC 33277 could invade PDLSCs after 2 h of incubation. However, endocytic vacuoles were not found surrounding the internalized *P. gingivalis* cells (Fig. 6b, c-a). Several bumps on the membrane of normal PDLSCs were observed, which were patches of stretched membrane where *P. gingivalis* ATCC 33277 had been being endocytosed (Fig. 6b, c-b).

## Discussion

Periodontitis is the main reason for tooth loss, and the regenerative capability of periodontal tissue is rather limited. Periodontal tissue repair is entirely dependent on implanted exogenous substitutes, though we are far from reaching the desired goal of the restoration of periodontal structure and function. The recent development of tissue engineering techniques has provided new opportunities for facilitating periodontal tissue regeneration, and the choice of seed cells is one of the key factors.

Stem cells have been a hot topic in medicine and biology in recent years and are of great importance to researchers. Adult stem cells have been found in almost all tissues, including bone marrow, cartilage, blood, nerves, muscle, fat, skin, and the cornea, intestine, liver and pancreas [22]. PDLSCs, a type of typical adult stem cell, can be obtained from the periodontal membrane. Numerous studies have shown that PDLSCs can proliferate, differentiate, and migrate to achieve tissue regeneration when periodontal tissue is repaired under pathological conditions or external damage. Therefore, PDLSCs are considered ideal seed cells for the treatment of periodontal defects [23]. However, to date, there are no standard criteria for the identification of PDLSCs [24]. PDLSCs are primarily identified according to their related properties, such as cloning capacity, multi-directional differentiation in vitro, and surface molecules.

Clone formation capacity reflects important cell characteristics such as dependencies and proliferation ability. The PDLSCs in our study were able to undergo clone-like growth, and the colony-forming efficiency was approximately 15.35%, slightly lower than previously reported [25]. We believe this difference might be associated with the cell growth status, tissue origin and culture conditions. The cytoskeleton provides a network structure in the cytoplasm that supports and maintains cell morphology and movement and is mainly composed of microfilaments, microtubules, intermediate filaments and the microtrabecular network. However, intermediate filaments differ among cells, such as keratin in epithelial cells, vimentin in mesenchymal cells and desminin muscle cells. These filaments are specific in chemical composition: they can be used as an antigen for the intermediate filament and thus be used to classify and identify cells. Immunohistochemical staining demonstrated the presence of vimentin but the absence of keratin in our PDLSCs, which indicated a mesenchymal, and not epithelial, origin for the cultured cells.

Cell growth and cell cycle status are basic parameters of cellular characteristics and are frequently used to evaluate cell viability. Previous studies have shown that the growth of PDLSCs is slower than that of other transitory proliferative cells [26]. Nevertheless, the multiplication rate of PDLSCs improved when the tissue was repaired. The growth curve of PDLSCs in our study exhibited an S-shaped curve: the cells began to grow rapidly after 4 days of incubation and reached a plateau on the 8th day. The PDLSCs showed typical cell cycle characteristics of stem cells, with most being in the/G1 phase (75.81%) and only a few in S phase (19.80%). These results showed that the proliferation of PDLSCs was slow.

The identification of markers on the stem cell surface is important and aids in the separation, identification and analysis of stem cells. However, specific markers for PDLSCs have not been reported. Previous studies have indicated that the surface markers of PDLSCs are similar to those of bone marrow mesenchymal stem cells [27, 28]. In our study, PDLSCs were positive for expression of fibroblast surface markers (CD29, CD90, CD105) and negative for hematopoietic markers (CD34, CD45). At the same time, the PDLSCs expressed the early molecular markers of mesenchymal stem cells (CD146, STRO-1).

Stem cells have the characteristics of self-replication and multi-directional differentiation capacity. Our studies have shown that PDLSCs can be differentiated into osteoblasts and lipoblasts under the appropriate circumstances. Indeed, the multi-directional differentiation capacity of PDLSCs has great potential for clinical application in periodontal regeneration.

In the present study, we succeeded in culturing and separating original periodontal ligament stem cells using the tissue block with limiting dilution method. Furthermore, we identified stem cell properties based on cell morphology, clone formation ability, growth activity, cell surface antigens and multiple differentiation capacities.

Plaque is an important factor in the occurrence and development of periodontal disease, and *P. gingivalis* has been confirmed to be closely related to periodontitis [29]. Accordingly, much research has verified that *P. gingivalis* can infect and invade a variety of host cell types in vitro, including primary human gingival epithelial cells [30], KB cells [31], endothelial cells [32], and gingival fibroblasts [33]. *P. gingivalis* has been detected in gingival tissues obtained from patients with periodontitis, indicating an essential effect of invasion in the pathogenesis of periodontitis [34]. Furthermore, the effects of *P. gingivalis* have also been studied in undifferentiated bone marrow stromal cells, in which *P. gingivalis* stimulates osteolytic cytokine expression production [35] via the p38 MAPK pathway [36], and activates a number of genes related to cell cycle arrest and apoptosis as confirmed by microarray analysis [37]. Previous studies showed that the invasion of *P. gingivalis* is a rapid process, reaching completion. Imaging of infected monolayers revealed that over 90% of gingival epithelial cells were invaded by *P. gingivalis* after 12 min [38]. Although the invasion efficiency did not increase after 2 h of incubation, when the interaction time was extended to 5 h, the number of internalized ecovered bacteria increased greatly because the bacteria divided within the epithelial cells [39]. Thus, in our study, we set a 2-h time point for observing PDLSCs infection by *P. gingivalis* ATCC 33277.

The traditional culture method is the classical approach for detecting bacterial invasion. Invasion rates of 10% and 30% have been reported for *P. gingivalis* in various cell types [40]. In our study, The PDLSC infection rates of *P. gingivalis* at MOIs of 50, 100, 200, and 500 were 5.83%, 8.12%, 7.77% and 7.53% according to the agar plate culture method. By q-PCR, the efficiencies of *P. gingivalis* infection of PDLSCs at MOIs of 50, 100, 200, and 500 were 6.74%, 10.56%, 10.36% and 9.78%, respectively. These results are similar to those of others. The infection efficiency according to the q-PCR method was higher than that according to the agar plate culture method. In addition, the difference between the agar plate culture and q-PCR methods was significant. It is not unexpected for differences between the results obtained from distinct methods. The culture assay uses intracellular survival as an alternative measure of infection and thus requires that the organisms remain viable throughout entire process. Furthermore, the culture method has a low sensitivity and is imprecise, and bacterial growth might affect the final results. The q-PCR amplification method not only has higher sensitivity than traditional culture techniques but is also less time consuming, enabling more accurate results within a shorter period of time. The optimal MOI for *P. gingivalis* ATCC 33277 was 100, which is similar to other results. We speculate, the bacteria have specific intake pathways at low MOIs and that at high MOIs, the cells were overwhelmed by the bacterial challenge via nonspecific mechanisms, or the interaction between *P. gingivalis* and PDLSCs may be related to saturation in surface receptors and signal transduction pathways.

The abilities to adhere to and invade host cells are important attributes of a successful pathogen. It has not been clearly elucidated how *P. gingivalis* enters in infected cells. Intracellular *P. gingivalis* reportedly localizes in various cellular compartments. *P. gingivalis* is localized in the perinuclear region of gingival epithelial cells [39] or in the cytoplasm in pocket epithelial cells [41]. In contrast, *P. gingivalis* is not observed in the cytoplasmic spaces but replicates in endocytic vacuoles of endothelial cells [42]. Additional studies are warranted to explain these differences, which are from the initial interactions between *P. gingivalis* and various types of host cells. In this study, the electron microscopic observations showed that *P. gingivalis* ATCC 33277 can invade PDLSCs, but the bacteria were found in the cytoplasm and were not encapsulated by endocytic vacuoles. Several bumps were patches of stretched membrane where *P. gingivalis* ATCC 33277 had been being endocytosed. So we suspect that the invasive properties of *P. gingivalis* in PDLSCs were similar to those in gingival epithelial cells. *P. gingivalis* exploits cellular endocytosis to enter cells, which is captured by cellular pseudopodia and invagination. Previous reports suggest that, following adhesion to the integrins, invasive event has been reported to require cytoskeleton [43], microtubules, integrin [44], lipid rafts [45] and bacterial fimbriae [46]. Microorganism invasion of host cells triggers signaling pathways that rearrange the cytoskeleton, facilitating bacterial entry and aiding their survival by avoiding extracellular degradation.

Therefore, *P. gingivalis* is able to enter PDLSCs in this study. Additional studies are required to better understand functional changes after bacterial invasion, such as differentiation and migration of PDLSCs, the intracellular lifestyle of *P. gingivalis* in PDLSCs.

## Conclusion

We succeeded in culturing and separating original periodontal ligament stem cells through the tissue block with limiting dilution method. *P. gingivalis* was able to invade periodontal ligament stem cells. The most optimal MOI for P*. gingivalis* ATCC 33277 was 100.

## Additional file

Additional file 1: Figure S1. The standard curve and amplification plot of 16S for *P. gingivalis* by q-PCR. (DOCX 154 kb)

## Abbreviations

BHI: Brain heart infusion
BSA: Bovine serum albumin
CFUs: Colony-forming units
DMEM: Dulbecco’s modified Eagle’s medium
FITC: Fluorescein isothiocyanate
MAPK: Mitogen-activated protein kinase.
MOI: Multiplicity of infection
*P. gingivalis*: *Porphyromonas gingivalis*
PBS: Phosphate-buffered saline
PDLSCs: Periodontal ligament stem cells
q-PCR: Quantitative polymerase chain reaction
