# Supplementary material for: Porphyromonas gingivalis can invade periodontal ligament stem cells
Source: BMC Microbiol. 2017 Feb 17;17:38. doi: 10.1186/s12866-017-0950-5 (PMC5316216; doi:10.1186/s12866-017-0950-5)
Supplement: Additional file 1: Figure S1. — The standard curve and amplification plot of 16S for P. gingivalis by q-PCR. (DOCX 154 kb) [file 12866_2017_950_MOESM1_ESM.docx]

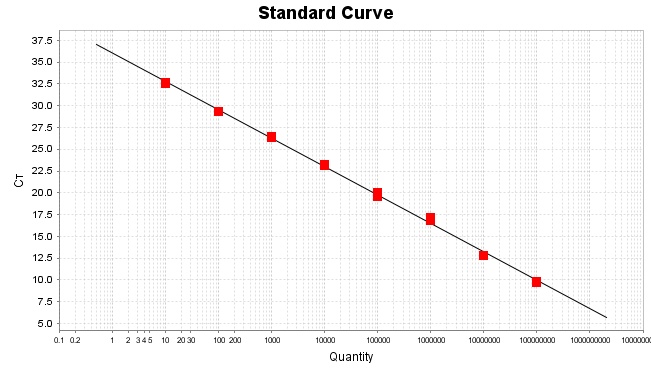


(a)


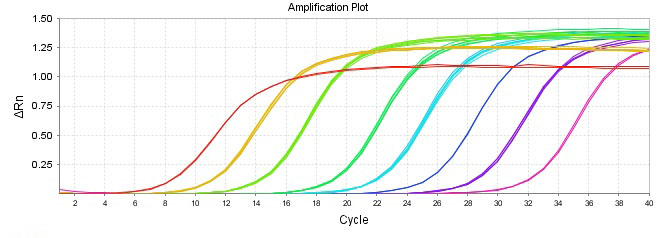


(b)

**Fig .1.** The standard curve and amplification plot of 16S for  *P. gingivalis* by q-PCR

A gradient dilution suspension of *P. gingivalis* ATCC 33277 from 10 to 10^8^/ml was prepared. After DNA was extracted, the q-PCR was carried out with primers specific of 16S for *P. gingivalis*. A standard curve (Y=39.569-3.43X) was generated (a). The amplification plot of 16S for *P. gingivalis* by q-PCR was showed (b).
